# Structural brain signatures of frailty, defined as accumulation of self-reported health deficits in older adults

**DOI:** 10.3389/fnagi.2023.1065191

**Published:** 2023-01-19

**Authors:** Raquel Gutiérrez-Zúñiga, James R. C. Davis, Kathy Ruddy, Céline De Looze, Daniel Carey, James Meaney, Rose Anne Kenny, Silvin Paul Knight, Roman Romero-Ortuno

**Affiliations:** ^1^Global Brain Health Institute, Trinity College Dublin, Dublin, Ireland; ^2^The Irish Longitudinal Study on Ageing, Trinity College Dublin, Dublin, Ireland; ^3^Discipline of Medical Gerontology, School of Medicine, Trinity College Dublin, Dublin, Ireland; ^4^Trinity College Institute of Neurosciences, Trinity College Dublin, Dublin, Ireland; ^5^St. James’s Hospital, Mercer’s Institute for Successful Ageing, Dublin, Ireland

**Keywords:** brain structure, frailty, brain health, ageing, magnetic resonance imaging

## Abstract

**Background:**

Frailty in older adults has been associated with reduced brain health. However, structural brain signatures of frailty remain understudied. Our aims were: (1) Explore associations between a frailty index (FI) and brain structure on magnetic resonance imaging (MRI). (2) Identify the most important FI features driving the associations.

**Methods:**

We designed a cross-sectional observational study from a population-based study (The Irish Longitudinal Study on Aging: TILDA). Participants aged ≥50 years who underwent the wave 3 MRI sub-study were included. We measured cortex, basal ganglia, and each of the Desikan-Killiany regional volumes. Age-and sex-adjusted correlations were performed with a 32-item self-reported FI that included conditions commonly tested for frailty in research and clinical settings. A graph theory analysis of the network composed by each FI item and cortex volume was performed. White matter fiber integrity was quantified using diffusion tensor imaging (DTI).

**Results:**

In 523 participants (mean age 69, 49% men), lower cortex and thalamic volumes were independently associated with higher FI. Sensory and functional difficulties, diabetes, polypharmacy, knee pain, and self-reported health were the main FI associations with cortex volume. In the network analysis, cortex volume had a modest influence within the frailty network. Regionally, higher FI was significantly associated with lower volumes in both orbitofrontal and temporal cortices. DTI analyses revealed inverse associations between the FI and the integrity of some association bundles.

**Conclusion:**

The FI used had a recognizable but subtle structural brain signature in this sample. Only some FI deficits were directly associated with cortex volume, suggesting scope for developing FIs that include metrics more specifically related with brain health in future aging neuroscience studies.

## Introduction

With progressively ageing societies and global preventative efforts being aimed at maintaining good physical function (Lancet, 2011) and brain health (Livingston et al., 2020) for as long as possible, the neuroscience of human ageing is a topic of increasing interest following emerging evidence that different lifespan exposures may affect brain ageing differently (Grady, 2012).

Frailty is an age-related state of physical vulnerability due to dysregulation in multiple physiological systems that is an important contributor to morbidity and mortality in older adults (Castellana et al., 2021; Bortone et al., 2022). Different ways to measure frailty have been proposed (de Vries et al., 2011; Howlett et al., 2021). Fried et al. (2001) proposed a definition of frailty based on 5 items (unexplained weight loss, weakness, low physical activity, slow walking speed, and exhaustion; Fried et al., 2001). Later, Rockwood and Mitnitski (2011) proposed to measure frailty as a composite of deficits that are related with age in a Frailty Index (FI). The FI sees frailty as the degree of accumulation of health deficits during the ageing process (Rockwood and Mitnitski, 2011), which as per standard procedure (Searle et al., 2008) can be composed by self-reported and/or test-based (Theou et al., 2015) symptoms, conditions, morbidities and/or disabilities that accumulate with age, cover a range of systems without saturating too early, and relate to adverse health outcomes. In 2013, the IANA/IAGG International Consensus Group proposed the definition of cognitive frailty as individuals with physical frailty and mild cognitive impairment with a clinical dementia rating score of 0.5 (Kelaiditi et al., 2013).

An emerging focus of enquiry is that frailty may be associated with risk of decline in brain health. This hypothesis is biologically plausible because ultimately, the brain is an organ of the body. However, in older adults, the evidence as to how frailty affects brain neuroimaging biomarkers is scarce and studies to date offer potentially contradictory results. On the one hand, with the frailty phenotype (FP) some authors found affectation of the brain microstructure (both gray and white matter; Maltais et al., 2020; Tian et al., 2020), with one study only finding association between the slowness component and cortex volume (Nishita et al., 2019). On the other hand, other authors using a 54-item FI that included the execution of instrumental activities (e.g., dressing, using the remote), chronic diseases (e.g., cardiovascular, malignancy) and sensory deficits (e.g., vision and hearing) did not find a relationship with white or gray matter volumes (Jordan et al., 2021). A group from Taiwan explored the impact of the physio-cognitive syndrome (physical slowness plus a score of >1.5 standard deviation from the mean for one cognitive domain) with changes in brain structure focusing on the hippocampus and the cerebellum (Chung et al., 2021). However, regardless of the definition of frailty, the mechanisms that may explain the relationship between frailty and brain health (e.g., neurological, cognitive, sensorimotor skills) are still unknown.

Our aim was to explore cross-sectional associations between a self-reported FI (including physical, sensory and cognitive features together with age-related chronic diseases) with gray matter volume (global cortex volume and basal ganglia) on magnetic resonance imaging (MRI) using data from The Irish Longitudinal Study on Ageing (TILDA). In addition, we aimed to identify the FI features that had a core role in the frailty-cortex volume relationship and, with a graph theory approach, attempt to better understand the relationship between each FI item and the cortex volume as a network of interconnected features. Finally, we aimed to identify brain regions (cortex regions and white matter tracts) associated with the FI.

## Materials and methods

### Participants

The Irish Longitudinal Study on Ageing is a prospective cohort study that collects health, economic, and social data from a nationally representative sample of community-dwelling adults living in Ireland aged 50 years and over (Kearney et al., 2011; Donoghue et al., 2018). We utilized data from participants in wave 3 of the study (March 2014–December 2015 cohort), which to date is the only wave where neuroimaging data was collected (Whelan and Savva, 2013; Donoghue et al., 2018). Of all participants attending the wave 3 health assessment center, a random subset was invited to return for multi-parametric brain MRI at the National Centre for Advanced Medical Imaging (CAMI) at St. James’s Hospital, Dublin, Ireland.^1^ Participants with self-reported history of stroke or transient ischemic attack (TIA) as well as those with neurodegenerative diseases (e.g., Parkinson’s Disease) were excluded for the purpose of this study. In addition, all MRI images were reviewed by an expert neuroradiologist from CAMI and any participants with structural brain lesions that could adversely affect the analysis (i.e., stroke, brain tumor) were excluded. Ethical approval was obtained from the Faculty of Health Sciences Research Ethics Committee in Trinity College Dublin, Ireland. Additional ethics approval was received for the MRI sub-study from the St. James’s Hospital/Adelaide and Meath Hospital, Inc. National Children’s Hospital, Tallaght (SJH/AMNCH) Research Ethics Committee, Dublin, Ireland. Written informed consent was obtained from all TILDA participants. Those attending for MRI also signed an additional MRI-specific consent form.

### Magnetic resonance imaging protocol

Participants were briefed on the protocol ahead of acquisition, which comprised a variety of scans including T1 3D Magnetization-prepared Rapid Gradient Echo (MP-RAGE) sequences and Diffusion Tensor Imaging (DTI) sequences. Scans were acquired *via* 3 T Philips Achieva system and 32-channel head coil. The acquisition parameters (T1-MP-RAGE and DTI) and protocols for the pre-processing and extraction of gray matter measures (cortical and subcortical) and white matter tracts are fully described in the Supplementary Appendix S1–S3. Briefly, T1 data pre-processing and extraction of brain volumes were automatically performed using Freesurfer v6.0 (Dale et al., 1999; Fischl et al., 1999a,b) and the associated pipelines and converted to cubic centimeters. The DTI pre-processing and data extraction were performed using Explore DTI (Leemans et al., 2009). All images were inspected for evidence of image artifact and presence of gray and white matter lesions by a trained operator blind to participant identity.

### Participant characteristics and frailty measure

Participant characteristics included age, sex, medical history (number of regular medications, vascular diseases, cancer, and smoking history), physical exercise in metabolic equivalents (METs; Donoghue et al., 2018) and quality of life measured by the CASP-12 scale (Wiggins et al., 2008).

Frailty was measured by a self-reported 32-item FI previously operationalized in TILDA (O’Halloran and O’Shea, 2017; Roe et al., 2017). For each participant, the FI was calculated as the sum of the deficits present divided by 32. The 32 items are described in the Supplementary Appendix S4. Of note, none of the included participants had a positive answer to the “stroke and transient ischemic attack” item. For descriptive purposes, FI scores of <0.10, 0.10–0.24, and ≥0.25 were used to classify participants as robust, pre-frail and frail, respectively (O’Halloran and O’Shea, 2017).

### Descriptive statistics

Statistical analysis was performed using IBM SPSS Statistics for Windows version 26 (SPSS Inc., Chicago, IL). Categorical variables were expressed as percentages and compared between groups using the Chi-square test or z-proportions where appropriate, with Bonferroni post-hoc adjustment. Continuous variables were expressed as mean and standard deviation (SD) and were compared using one-way ANOVA test for multiple comparisons with least significant different (LSD) post-hoc analysis. All tests were two-tailed, and statistical significance was defined as *p* < 0.05. For the partial correlation models, we decided not use a multiple comparison correction, as recommended for the exploratory design of this study (Rothman, 1990).

### Frailty and global brain volumes

To explore the association between FI and global brain volumes, we first tested the association between FI and total cortex volume with a partial correlation adjusted by age and sex; and with the FI and the total basal ganglia volumes (caudate, putamen and thalamus, left and right separately) also adjusted by age and sex. We then explored which items within the FI were most closely related with volumes, by performing partial correlation analyses with the cortex volume and each one of the items of the FI, adjusted by age and sex.

To study how the FI items and the cortex volume interacted with each other, we constructed an adjacency matrix from a correlation matrix (adjusted by age and sex) assigning 1 to significant associations and 0 to non-significant associations with a threshold of *p* < 0.05 (Batushansky et al., 2016). Building a network from a correlation matrix for the FI features and cortex volume allowed us to analyze the network properties, for example its degree (total number of connections), betweenness centrality (the fraction of all shortest paths in the network that pass through a given node; Rubinov and Sporns, 2010) and modularity (densely interconnected nodes within each module of the network; Bullmore and Sporns, 2009). The degree, betweenness centrality and modularity were calculated with Gephi version 9.4 (Bastian et al., 2009) as a non-directed graph with the in-built algorithm for modularity partition (Blondel et al., 2008).

### Frailty and regional brain volumes

Regions of interest (ROIs) cortex volumes were based on the Desikan-Killiany atlas parcellation (Desikan et al., 2006). To explore which regional brain volumes were associated with the FI, we constructed partial correlation models adjusted by age, sex, and the total cortex volume.

### Frailty and white matter integrity

To explore the association between the FI and white matter integrity, we performed a partial correlation analysis of fractional anisotropy (FA) and mean diffusivity (MD) with the FI, adjusted by age and sex. We only considered a relationship between FI and white matter integrity if both measures (FA and MD) were correlated with it (Madden et al., 2012).

## Results

**Figure 1:**
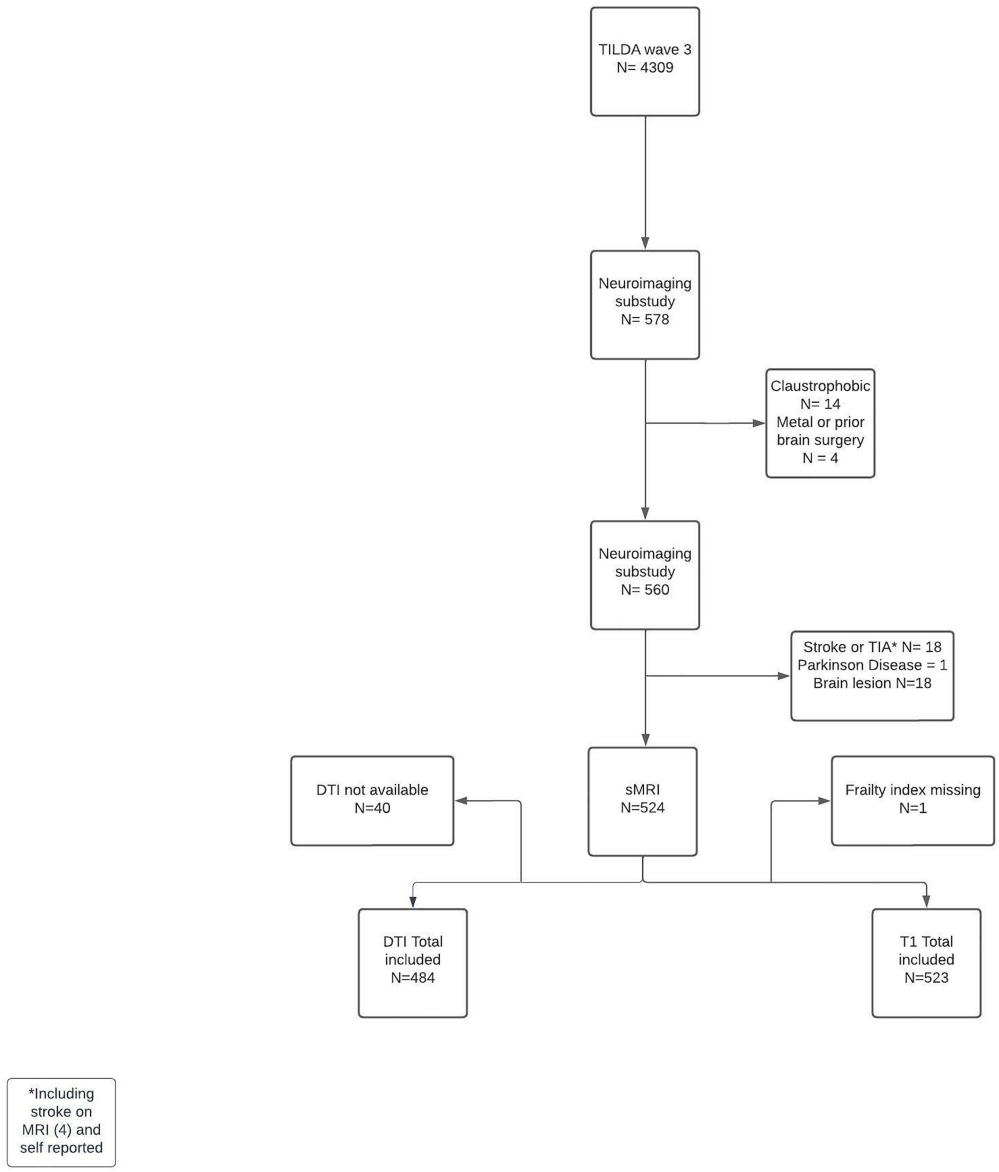
Participants’ flow chart. TIA, transient ischaemic attack; DTI, diffusor tensor imaging; sMRI, structural MRI.

A total of 523 participants were included in the brain volumes analyses, and 484 had data for white matter integrity analyses. The flowchart of included participants and full exclusion details is shown in Figure 1. The characteristics of the 523 participants (overall and by FI group) are described in Table 1. Overall, the proportion of frailty in this sample was low (7.3%, *n* = 38). Frailer participants were older, had higher prevalence of vascular diseases, were taking a higher number of medications, and reported lower physical activity levels and lower overall quality of life.

**Table 1:** Participants’ characteristics.

	Robust (FI <0.10) *N* = 295	Pre-frail (FI 0.10–0.24) *N* = 190	Frail (FI ≥0.25) *N* = 38	*p*	Overall *N* = 523
Male; N (%)	152 (51.9)	87(45.8)	15 (39.5)	0.237	254 (48.6)
Age (years); mean (SD)	66.9 (7.0)*	70.7 (7.0)*	74.3 (6.9)*	<0.001	68.8 (7.3)
Number of medications; mean (SD)	1.3 (1.3)*	3.2 (2.2)*	6.0 (3.2)*	<0.001	2,85 (2.4)
Hypertension; N (%)	63 (21.4)*	97 (51.1)	22 (57.9)	<0.001	182 (34.8)
Diabetes; N (%)	9 (3.1)*	28 (14.7)	10 (26.3)	<0.001	47 (9.0)
High cholesterol; N (%)	72 (24.4)*	91 (47.9)*	28 (73.7)*	<0.001	191 (36.5)
BMI; mean (SD)	27.7 (4.5)*	28.3 (4.6)	29.5(5.3)*	0.051	28 (4.6)
Cancer; N (%)	1 (0.3)*	16 (8.4)	5 (22.7)	<0.001	22 (4.2)
Atrial fibrillation, irregular heart rhythm; N (%)	5 (1.7)*	11 (5.8)	2 (11.1)	0.044	18 (3.4)
Stroke; N (%)	0	0	0	-	0
Difficulty walking 100 m; N (%)	0	1 (0.5)*	7 (18.4)*	<0.001	8 (1.5)
Poor self-rated physical health; N (%) Fair Poor	2 (0.7)* 0*	21 (11.1)* 1 (0.5)	11 (28.9)* 2 (5.3)*	<0.001	34 (6.5) 3 (0.6)
Poor self-rated vision; N (%) Fair Poor	5 (1.7)* 1 (0.3)*	14 (7.4) 4 (2.1)	5 (13.2)* 3 (7.9)*	<0.001	24 (4.6) 8 (1.5)
Poor self-rated hearing; N (%) Fair Poor	24 (8.1)* 2 (0.7)*	34 (17.9)*^†^ 5 (2.6)	12 (31.6)^†^ 2 (5.3)*	<0.001	70 (13.4) 9 (1.7)
Polypharmacy; N (%)	8 (2.7)*	62 (32.6)*	29 (76.3)*	<0.001	99 (18.9)
Knee pain; N (%)	7 (2.4)*^†^	16 (8.4)*	8 (21.1) ^†^	<0.001	31 (5.9)
Difficulty following a conversation with four people; N (%) Some Yes	43 (14.6)*^†^ 6 (2)*	57 (30)* 12 (6.3)*	14 (36.8) ^†^ 8 (21.1)*	<0.001 <0.001	114 (21.8) 26 (5)
Difficulty kneeling; N (%)	26 (8.8)*	74 (38.9)*	34 (89.5)*	<0.001	134 (25.6)
Difficulty reaching above shoulder; N (%)	2 (0.7)*^†^	23 (12.1)*	12 (31.6)^†^	<0.001	37 (7.1)
Urinary incontinence; N (%) Some Yes	9 (3.1) 9 (3.1)*^†^	8 (4.2) 22 (11.6)*	3 (7.9) 9 (23.7)^†^	>0.05 <0.001	20 (3.8) 40 (7.6)
Daytime sleepiness; N (%) Moderate chance High chance	41 (13.9)* 31 (10.5) *^†^	43 (22.6)* 39 (20.5)*	8 (21.1) 14 (36.8)^†^	<0.001 <0.001	92 (17.6) 8 (16.1)
Angina; N (%)	0^†^	3 (1.6)*	7 (18.4)*^†^	<0.001	10 (1.9)
Hearth attack; N (%)	0	1 (0.5)	0	–	1 (0.2)
Difficulty rising from a chair; N (%)	10 (3.4)*	37 (19.5)*	20 (52.6)*	<0.001	67 (12.8)
Other CVD; N (%)	0	3 (1.6)	1 (2.6)	0.058	4 (0.8)
Cataracts; N (%)	14 (4.7)*	43 (2.6)	13 (34.2)	<0.001	70 (13.4)
Arthritis; N (%)	61 (20.7)*	101 (53.2)*	30 (78.9)*	<0.001	192 (36.7)
Osteoporosis; N (%)	29 (9.8)*	41 (21.6)	13 (34.2)	<0.001	83 (15.9)
Varicose ulcer; N (%)	5 (1.7)	3 (1.6)	2 (5.3)	0.292	10 (1.9)
Difficulty climbing one flight of stairs; N (%)	1 (0.3)	5 (2.6)	12 (31.6)*	<0.001	18 (3.4)
Glaucoma/ARMD; N (%)	6 (2)*	20 (10.5)	7 (18.4)	<0.001	33 (6.3)
Day to day memory; N (%) Fair Poor	31 (10.5)* 3 (1)*	48 (25.3)* 3 (1.6)	13 (34.2) 3 (7.9)*	<0.001 <0.001	92 (17.6) 9 (1.7)
Difficulty pushing objects; N (%)	4 (1.4)*	17 (8.9)*	17 (44.7)*	<0.001	38 (7.3)
Difficulty lifting weights; N (%)	4 (1.4)*	32 (16.8)*	23 (39)*	<0.001	59 (11.3)
Difficulty picking up coins; N (%)	0*	9 (4.7)*	6 (15.8)*	<0.001	15 (2.9)
Feeling lonely; N (%) Some Yes	11 (3.7)* 0	17 (8.9)*^†^ 7 (3.7)	4 (10.5)*^†^ 0	0.001 –	32 (6.1) 7 (1.3)
Education; N (%): - None/primary - Secondary - Third/higher	49 (16.6)* 110 (37.3)* 136 (46.1)*	50 (26.6) 62 (32.6)* 78 (41.1)*	12(31.6) 16 (42.1)* 10 (26.3)*	0.020	111 (21.2) 188 (35.9) 224 (42.8)
Current smoker; N (%)	11 (5.2)	6 (3.2)	3 (6.1)	0.391	20 (4.5)
MET; mean (SD)	3931.1 (5679.8)*	2687.5 (4166.2)*	1439.1 (2696.9)*	0.002	3,294 (5052.1)
CASP-12 autonomy; mean (SD)	6.8 (1.7)*	6.3 (1.6)*	4.9 (1.6)*	<0.001	6.5 (1.7)
CASP-12 control; mean (SD)	8.8 (2.2)*	8.0 (2.1)*	6.2 (1.8)*	<0.001	8.3 (2.2)
CASP-12 pleasure; mean (SD)	8.2 (1.7)	8.3 (1.2)	8.2 (1.2)	0.506	8.2 (1.5)
CASP-12 self-realization; mean (SD)	5.1 (1.3)	5.1 (1.1)	4.8 (1.1)	0.462	5.1 (1.2)
CASP-12 QoL; mean (SD)	28.9 (5.0)*	27.7 (4.7)*	24.2 (4.3)*	<0.001	28.1 (5)

*Groups where proportions were significantly different from the rest; ^†^Bonferroni adjustment. BMI, body mass index; MET, metabolic equivalent of task; CVD, cardiovascular diseases; ARMD, age related macular degeneration; CASP, control, autonomy, self-realization, pleasure scale; QoL, quality of life.

### Frailty index and global brain volumes

**Figure 2:**
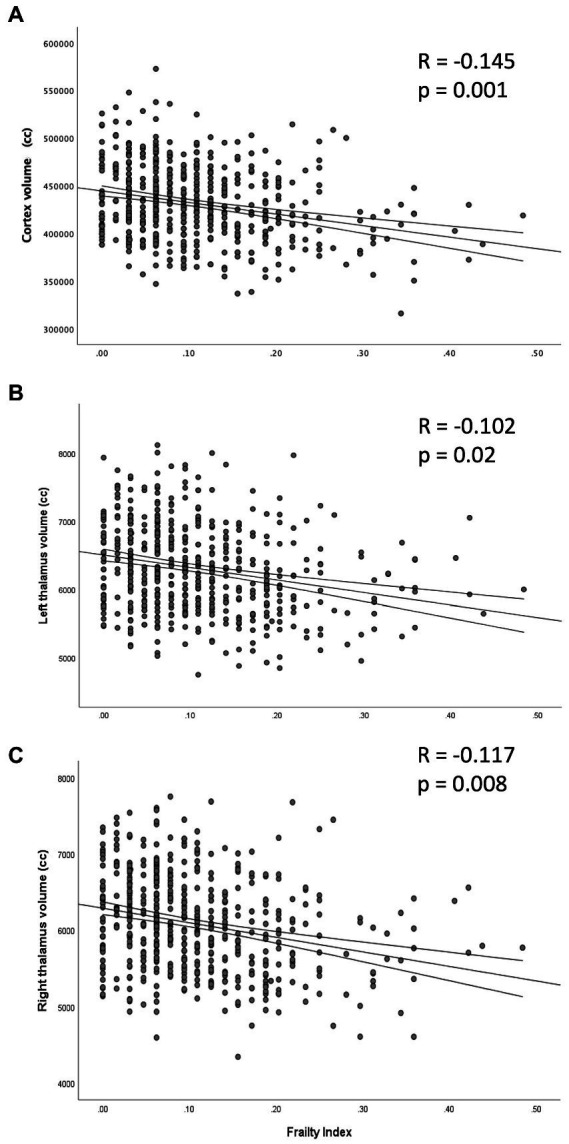
Partial correlation between the frailty index and the volume of cortex **(A)** and thalami (**B**: left; **C**: right), adjusted by age and sex.

Overall, the FI was negatively correlated with the cortex volume, meaning that the frailer a participant was, the more global cortex atrophy was present; and this relationship was independent of age and sex (Figure 2A). For the basal ganglia, both thalamic volumes were negatively correlated with the FI (Figures 2B, C).

**Figure 3:**
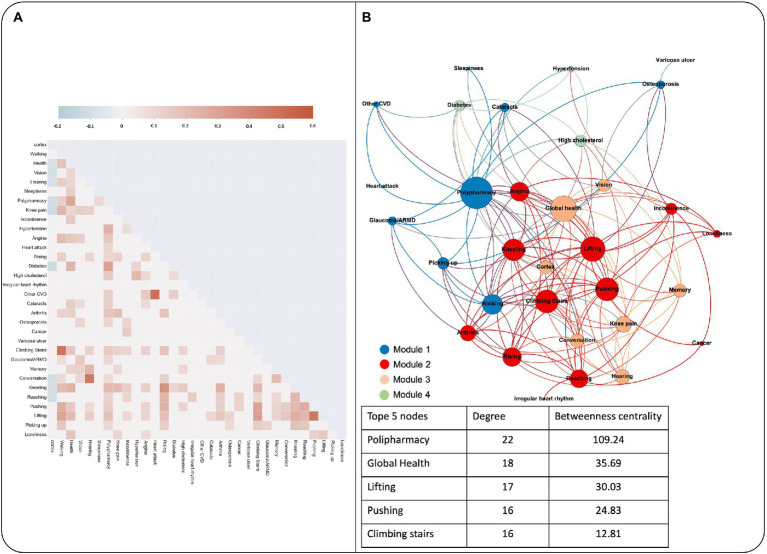
**(A)** Heatmap from the correlation matrix that shows the significant correlations between cortex volume and the FI items among them. **(B)** Graph theory analysis of the network composed by each FI item and cortex volume. Each module is depicted in a different color, and each node that it is included in the module is colored according to it. The node size is proportional to the degree. The table shows the top five most connected nodes, with the degree and betweenness centrality values.

Figure 3A shows the significant correlations found for items within the FI and cortex volume. The first column correspond to the correlations with the cortex volume. Only 9 of the 32 items, namely diabetes, functional difficulties (i.e., kneeling, reaching objects), general health (poor self-reported health, knee pain, and polypharmacy) and sensory deficits (poor vision, hearing, and difficulty following a conversation) were correlated with total cortex volume.

When all the items of the FI and the cortex volume were analyzed as a graph to see each other’s interactions, we found that the graph distribution could be organized within 4 modules of highly associated nodes (Figure 3B). The highest degree nodes within the network were polypharmacy, global health perception and functional difficulties, whereas the cortex volume did not reach the top 5-degree nodes (15th position within the network, 9 degree and 7.37 betweenness centrality).

### Frailty and regional brain volumes

**Figure 4:**
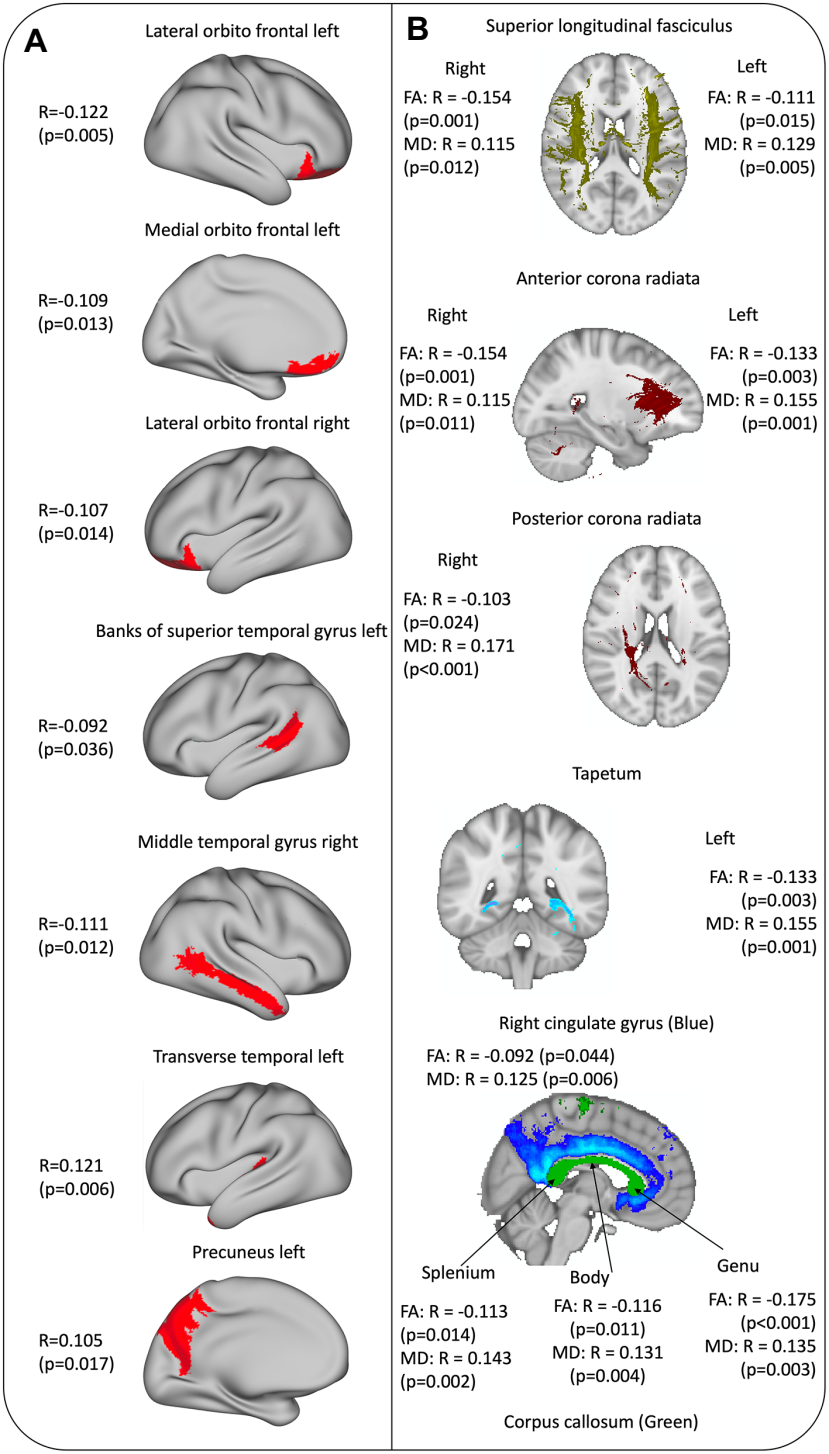
**(A)** Cortex brain areas associated with frailty. **(B)** White matter tracts associated with frailty.

Results are presented in Figure 4A. Lower volumes with higher frailty were seen in hubs of high-order information integration in frontal and temporal lobes; whereas there was a positive correlation between the FI and volumes in the left precuneus and left transverse temporal gyrus.

### Frailty and white matter integrity

Results are presented in Figure 4B. With higher FI, there was lower fiber integrity (decrease in FA and increase in MD) in corpus callosum, and superior longitudinal fasciculus and anterior corona radiata in both hemispheres. In addition, with higher FI there was lower integrity in the right posterior corona radiata and right cingulate cortex, but this was not seen in the homologous tracts on the left hemisphere (Figure 4B).

## Discussion

The aim of this study was to explore cross-sectional associations between a self-reported FI and brain structure (global and regional brain volumes, and white matter integrity) using TILDA data. In addition, we aimed to identify which FI deficits had a core role in these associations. We found that an FI composed by self-reported chronic conditions and sensory and functional impairments was independently but weakly associated with reduced global cerebral cortex volume and reduced volumes in hubs of high-order information integration in frontal and temporal lobes, as well as thalamic volumes and white matter tracts’ integrity.

We found that the cerebral cortex volume and both thalamic volumes were associated with frailty independently of age and sex. Previous studies with different ways to measure frailty (e.g., physical frailty, Edmonton Frailty Scale) pointed to similar results (Del Brutto et al., 2017; Kant et al., 2018), so it is possible that these signals are a non-specific reflection of accelerated brain ageing that is commonly captured by different frailty operationalizations. From here, we went one step further and explored which FI deficits had the most important role in relation with neuroimaging biomarkers such as the cortex volume. The concept of network physiology emerged recently to highlight the fact that the functioning of different systems is interconnected (Romero-Ortuño et al., 2021). For this reason, we decided to use a graph theory approach to explore the interconnection between cortex volume and each of the FI features. To our knowledge, a graph theory approach had not been previously applied to understand the interrelationships between body and brain aspects of frailty. Previously, individual network models had been used to explore the connectivity among health deficits and mortality, observing that the transition from health to impairment in one characteristic depended on the state of the neighbor nodes, with damage propagating through a complex network of interconnected elements (Mitnitski et al., 2017). In our study, the cross-sectional design did not allow to establish causal relationships but allowed to visually depict the interconnection between the FI items and an objective structural brain measure. We observed that only 9 FI items were negatively associated with cortex volume. Moreover, the cortex volume did not achieve a core role within the network in terms of connectivity or centrality, suggesting that overall, our FI may have modest relevance to structural brain biomarkers. When we analyzed the modules within the network, one of the four modules contained the cortex volume as a node, again associated with a cluster of global health perception, and sensory and memory difficulties. Memory was not directly correlated with the cortex volume but was part of the module of highly connected features including cortex volume. More clearly, the modularity analysis showed that the items directly related with cortex volume were those related with sensory and cognitive deficits and global health perception, with low impact of cardiovascular diseases. However, other aspects not directly related with cortex volume had influence on the items that finally affected the cortex volume (e.g., difficulty lifting weights and angina influenced global health, and global health influenced cortex volume). Observing the landscape in this way could facilitate the future creation of an FI that is more specifically oriented to the study of brain health in ageing to find the health and functional conditions that have a significant impact on brain structure.

To further our results, we looked for specific brain regions related with our definition of frailty within the cortex, and we found that the orbitofrontal cortex and the regions in lateral temporal cortex in both hemispheres showed lower volumes with higher frailty independently of age and sex. Previous studies found that there are brain cortex areas that have a higher rate of atrophy than the rest of the brain during normal ageing. These areas include the orbitofrontal cortex, temporal pole and fusiform gyrus (Fjell et al., 2009). Moreover, some of these brain areas exhibit higher atrophy in relation with the accelerated “Pace of Ageing," an index constructed by Elliott et al. (2021) composed by different biomarkers present in younger adults (the orbitofrontal cortex, middle temporal cortex and insula). Interestingly in their study, participants with an accelerated pace of ageing also had higher sensory difficulties and a negative perception of their own health, similarly to the FI items that were related with cortex volume and brain regions in our sample. Furthermore, Tian et al. (2020) found that the medial frontal cortex, anterior cingulate cortex and basal ganglia volumes were reduced in frail older people (where frailty was characterized by the physical FP) independently of chronological age. Integrating all the information together, our results agree with previous studies that used a whole brain structure approach (but with different frailty measures) and point toward the orbitofrontal cortex and middle temporal gyrus as regions that may be implicated in frailty. It could be possible that to maintain a good functionality in ageing there must be an optimal integration among sensory-motor hubs in the central nervous system; and frailty (measure by physical and functional component) could be a reflection or a cause of this “disintegration.” This hypothesis should be supported by a functional brain connectivity analysis as well as other cohorts.

When looking at the results obtained for white matter integrity, we found that tracts that connect both hemispheres (corpus callosum) but mostly both frontal lobes (genu) and the frontal lobes with the rest of brain regions (anterior corona radiata with thalamus and superior longitudinal fasciculus with the rest of lobes) had higher affectation with frailty. This could point to the same conclusion in which the connection between regions should be optimal to have a good global physical functionality. However, the division of the association tracts made for this study is based on the anatomical definition, and we cannot assure that the cortex areas pointed were connected only or specifically by these tracts. However, those are important and coherent findings that should be further explored in future studies with functional connectivity analysis to see if there is a real disruption in the brain network.

We also found that the left precuneus and the left transverse superior gyrus volumes were positively correlated with frailty; and that only right posterior corona radiata and cingulate bundle integrity were negatively related with the FI. We could hypothesise that with our definition of frailty, the frontal cortices, and the connectivity from and in between them could be affected together with a relative preservation of the left posterior integration-association cortex and bundles, that perhaps is an adaptation of the brain with the accumulation of difficulties, especially when the sample used was extracted from a population-based sample and only 7% of participants were categorized as frail.

The main strengths of our research are: (a) the integration of different structural MRI modalities to study the relationship between a FI and brain health; (b) the network analysis to disentangle the main features related with the FI and brain structure; and (c) the identification of possible targets within the cortex volume that can be selected as regions of interest for further analysis.

Limitations of our study include: (a) the cross-sectional design does not allow us to disentangle if the findings are the cause of the deficits or the consequence of the accumulation of the health deficits and are indeed the reflection of a brain adaptation process; (b) all the deficits in our FI were self-reported, and a previous TILDA study highlighted variability in the participants’ responses over time and some disagreement across different surveys for the same participants (Romero-Ortuño et al., 2021); (c) the items included in the TILDA FI were not specifically chosen as possible indicators of brain health; (d) the analysis of brain structure with a broad pre-constructed parcellation could be underpowered to detect subtle deficits as we were studying a relatively healthy community-based sample; e) the correlations found for the structural MRI measures and the FI were low, which can be explained by the intrinsic characteristics of the sample (healthy population with only a 7% of participants classified as frail) and the partial correlation model used. An analysis of functional MRI could be useful to explore if there might be a difference in the high order network organization depending on the frailty status before structural changes occur that can affect integration brain areas such as orbitofrontal cortex or medial temporal cortex.

In conclusion, frailty defined as self-reported accumulation of health deficits had a subtle but recognizable signature on brain structure (high order integration areas and association white matter tracts mostly involving frontal lobes with relative preservation of left parietal cortex). The features that composed the FI had low weight vis-à-vis total cortex volume when their interactions were analyzed as a network. Future replication studies are required, ideally with longitudinal approaches; and refined frailty operationalization with a more specific focus on brain health are needed for future studies in the field of ageing neuroscience.

## Data availability statement

The datasets presented in this article are not readily available because TILDA provides access to the datasets for research use through anonymized publicly accessible dataset files, and through an on-site Hot Desk Facility. The publicly accessible dataset files are hosted by the Irish Social Science Data Archive based in University College Dublin, and the Interuniversity Consortium for Political and Social Research (ICPSR) based in the University of Michigan. Researchers wishing to access the data must complete a request form, available on either the ISSDA or ICPSR website. Requests to access the datasets should be directed to https://tilda.tcd.ie/data/accessing-data/.

## Ethics statement

The studies involving human participants were reviewed and approved by Faculty of Health Sciences Research Ethics Committee in Trinity College Dublin, Ireland. St. James’s Hospital/Adelaide and Meath Hospital, Inc., National Children’s Hospital, Tallaght (SJH/AMNCH) Research Ethics Committee, Dublin, Ireland. The patients/participants provided their written informed consent to participate in this study.

## Author contributions

RG-Z: conceptualization, data curation, formal analysis, investigation, methodology, validation, visualization, and writing-original draft. JRCD: writing-review and editing. KR: investigation, methodology, software, data curation, and writing-review and editing. CD and DC: investigation, methodology, and writing-review and editing. JM: data curation, investigation, methodology, resources, validation, visualization, and writing-review and editing. RK: funding acquisition, investigation, project administration, resources, and writing-review and editing. SK: data curation, investigation, supervision, and writing-review and editing. RR-O: conceptualization, funding acquisition, investigation, methodology, project administration, resources, supervision, and writing-review and editing. All authors agreed to the final version.

## Funding

TILDA was funded by the Irish Department of Health, Atlantic Philanthropies, and Irish Life. RG-Z and RR-O were funded by the Global Brain Health Institute, Trinity College Dublin. RR-O was also funded by a grant from Science Foundation Ireland 18/FRL/6188.

## Conflict of interest

The authors declare that the research was conducted in the absence of any commercial or financial relationships that could be construed as a potential conflict of interest.

## Publisher’s note

All claims expressed in this article are solely those of the authors and do not necessarily represent those of their affiliated organizations, or those of the publisher, the editors and the reviewers. Any product that may be evaluated in this article, or claim that may be made by its manufacturer, is not guaranteed or endorsed by the publisher.

## Footnotes

^1^ https://www.3tcentre.com/
