# Supplementary material for: Structural brain signatures of frailty, defined as accumulation of self-reported health deficits in older adults
Source: Front Aging Neurosci. 2023 Jan 19;15:1065191. doi: 10.3389/fnagi.2023.1065191 (PMC9892944; doi:10.3389/fnagi.2023.1065191)
Supplement: Supplementary file 1 [file Data_Sheet_1.docx]

**Supplementary material**

1. Appendix 1: MRI protocol.

2. Appendix 2: T1 MP-RAGE sequence pre-processing and data extraction.

3. Appendix 3: DTI pre-processing and data extraction.

4. Appendix 4: FI items.

5. References.

**1. MRI protocol.**

Participants were briefed on the protocol ahead of acquisition, which comprised a variety of scans including T1 and DTI sequences. Scans were acquired via 3T Philips Achieva system and 32-channel head coil. For the T1 3D Magnetisation-prepared Rapid Gradient Echo (MP-RAGE) sequence the acquisition parameters were: FOV (mm): 240 x 240 x 162; voxel size (mm): 0.8x0.8x0.9; SENSE factor: 2; TR: 6.7 ms; TE: 3.1 ms; flip angle: 8°. For DTI echo planar imaging (-EPI) 2D sequence the acquisition parameters were: FOV (mm) (244x244x140); voxel size (mm): 1.9 mm; SENSE factor 2; TR: 12887 ms; TE: 55 ms; flip angle: 90°.

**2. MRI pre-processing T1 MP-RAGE sequence**

All T1-weighted images were analysed using FreeSurfer v.6.0, with the associated cross-sectional pipeline to derive Regions of Interest (ROIs) in each subject’s native space [1,2,3], using the Desikan–Killiany atlas [4].

Briefly, the processing stream included motion correction and averaging of volumetric T1-weighted images, skull stripping, automated Talairach transformation, segmentation of the subcortical white matter (WM) and deep grey matter (GM) volumetric structures, non-uniform intensity normalisation, tessellation of the boundary between GM and WM, automated topology correction, and surface deformation. From this, data summaries for cortical regions were computed, including regional volume, surface area, curvature, and thickness [1,2,3]. In addition, the total brain volume was calculated excluding cerebrospinal fluid (ventricles), brainstem and choroid plexus.

All unprocessed input volumes were inspected for evidence of image artifact and presence of grey and white matter lesions by a trained operator blind to participant identity, using FreeView. Also, using Freeview, blinded inspections of all white matter and pial surfaces overlaid on the intensity normalised T1-weighted volume were also conducted for each participant.

Finally, for each participant, the total brain volume, subcortical and cortical grey matter volumetric measures (cm^3^) and for the ROIs as defined by the Desikan-Killiany atlas (68 cortical parcellations) were obtained from FreeSurfer segmentation statistical output (aseg) and then converted to cubic centimetre (cm^3^).

All procedures were run using batch processes on a Linux computing cluster via the Trinity Centre for High Performance Computing (https://www.tchpc.tcd.ie/).

**3. DTI pre-processing**

Data were processed using ExploreDTI [5]. Images were corrected for head movement and eddy currents using the procedure described in Leemans and Jones [6]. Tensor estimation was performed using the iteratively reweighted linear least squares approach [7]. Fibre trajectories were computed with CSD based tractography [8] using recursive calibration of the response function to optimise the estimation of the fibre orientation distribution (FOD) functions [9]. A uniform grid of tractography seed points at a resolution of 2x2x2 mm^3^ was used with an angle threshold of 30 degrees, an FOD threshold of 0.1, and maximum harmonic order of eight. The median number of streamlines computed for each participant was 55,221 (IQR 8665). Reconstructed fibre trajectories for each individual were quantified in terms of the (median) fractional anisotropy (FA) and mean diffusivity (MD) which are all measures that reflect the directional coherence of intracellular water diffusion. These were computed for the association with white matter tracts based on the definition in the ICBM-81 atlas [10].

**4. Frailty Index items**

The FI was calculated for each participant summing the total score and divided by 32 [11]. First and fourth columns are the denomination of the index for figures references.

| **Walking** | **Difficulty walking 100m** | Yes (1 point)  No (0 points) | **Cataracts** | **Cataracts** | Yes (1 point)  No (0 points) |
| --- | --- | --- | --- | --- | --- |
| **Global Health** | **Poor self-rated physical health** | Yes (1 point)  Some (0.5 points)  No (0 points) | **Arthritis** | **Arthritis** | Yes (1 point)  No (0 points) |
| **Vision** | **Poor self-rated vision** | Yes (1 point)  Some (0.5 points)  No (0 points) | **Osteoporosis** | **Osteoporosis** | Yes (1 point)  No (0 points) |
| **Hearing** | **Poor self-rated hearing** | Yes (1 point)  Some (0.5 points)  No (0 points) | **Cancer** | **Cancer** | Yes (1 point)  No (0 points) |
| **Sleepiness** | **Daytime sleepiness** | Yes (1 point)  Some (0.5 points)  No (0 points) | **Varicose Ulcer** | **Varicose ulcer** | Yes (1 point)  No (0 points) |
| **Polypharmacy** | **Polypharmacy (more than 5 regular medications)** | Yes (1 point)  No (0 points) | **TIA** | **Transient ischemic attack (TIA)** | Yes (1 point)  No (0 points) |
| **Knee pain** | **Knee pain** | Yes (1 point)  No (0 points) | **Climbing** | **Difficulty climbing one flight of stairs** | Yes (1 point)  No (0 points) |
| **Incontinence** | **Urinary incontinence** | Yes (1 point)  Some (0.5 points)  No (0 points) | **Glaucoma/ARMD** | **Glaucoma/ Age-related macular degeneration** | Yes (1 point)  No (0 points) |
| **Hypertension** | **Hypertension** | Yes (1 point)  No (0 points) | **Memory** | **Self-rated day to day memory** | Yes (1 point)  Some (0.5 points)  No (0 points) |
| **Angina** | **Angina** | Yes (1 point)  No (0 points) | **Conversation** | **Difficulty following a conversation with 4 people** | Yes (1 point)  Some (0.5 points)  No (0 points) |
| **Heart attack** | **Heart attack** | Yes (1 point)  No (0 points) | **Kneeling** | **Difficulty stooping, kneeling, or crouching** | Yes (1 point)  No (0 points) |
| **Rising** | **Difficulty rising from a chair** | Yes (1 point)  No (0 points) | **Reaching** | **Difficulty reaching above shoulder height** | Yes (1 point)  No (0 points) |
| **Diabetes** | **Diabetes** | Yes (1 point)  No (0 points) | **Pushing** | **Difficulty pushing/pulling large objects** | Yes (1 point)  No (0 points) |
| **Cholesterol** | **High cholesterol** | Yes (1 point)  No (0 points) | **Lifting** | **Difficulty lifting/carrying weights >= 10lb** | Yes (1 point)  No (0 points) |
| **Irregular heart rhythm** | **Irregular heart rhythm** | Yes (1 point)  No (0 points) | **Picking** | **Difficulty picking up coin from table** | Yes (1 point)  No (0 points) |
| **Other CVD** | **Other cardiovascular diseases** | Yes (1 point)  No (0 points) | **Loneliness** | **Feeling lonely** | Yes (1 point)  Some (0.5 points)  No (0 points) |

**5. References**

**1**. Dale AM, Fischl B, Sereno MI. Cortical Surface-Based Analysis: I. Segmentation and Surface Reconstruction. Neuroimage 1999; 9: 179–194.

**2**. Fischl B, Sereno MI, Dale AM. Cortical surface-based analysis. II: Inflation, flattening, and a surface-based coordinate system. Neuroimage 1999; 9: 195–207.

**3**. Fischl B, Sereno MI, Tootell RB, Dale AM. High-resolution intersubject averaging and a coordinate system for the cortical surface. Hum Brain Mapp 1999; 8: 272–284.

**4**. Desikan RS, Ségonne F, Fischl B *et al.* An automated labeling system for subdividing the human cerebral cortex on MRI scans into gyral based regions of interest. Neuroimage 2006; 31: 968–980.

**5**. Leemans A, Jeurissen B, Sijbers J, Jones DK. ExploreDTI: a graphical toolbox for processing, analyzing, and visualizing diffusion MR data. Proc Int Soc Magn Reson Med 2009; 17: 3537.

**6**. Leemans A, Jones DK. The B-matrix must be rotated when correcting for subject motion in DTI data. Magn Reson Med 2009; 61: 1336–1349.

**7**. Veraart J, Sijbers J, Sunaert S, Leemans A, Jeurissen B. Weighted linear least squares estimation of diffusion MRI parameters: strengths, limitations, and pitfalls. Neuroimage 2013; 81: 335–346.

**8**. Tournier J-D, Calamante F, Connelly A. Robust determination of the fibre orientation distribution in diffusion MRI: non-negativity constrained super-resolved spherical deconvolution. Neuroimage 2007; 35: 1459–1472.

**9**. Tax CMW, Jeurissen B, Vos SB, Viergever MA, Leemans A. Recursive calibration of the fiber response function for spherical deconvolution of diffusion MRI data. Neuroimage 2014; 86: 67–80.

**10**. Mori S, Oishi K, Jiang H *et al.* Stereotaxic white matter atlas based on diffusion tensor imaging in an ICBM template. Neuroimage 2008; 40: 570–582.

**11**. O’Halloran A, O’Shea M. Wellbeing andHealth in Ireland’s over 50’s 2009-2016 Frailty Chapter 7 TILDA. 2017; 117–134.
